# Microfabrication Process Development for a Polymer-Based Lab-on-Chip Concept Applied in Attenuated Total Reflection Fourier Transform Infrared Spectroelectrochemistry

**DOI:** 10.3390/s23146251

**Published:** 2023-07-08

**Authors:** Noah Atkinson, Tyler A. Morhart, Garth Wells, Grace T. Flaman, Eric Petro, Stuart Read, Scott M. Rosendahl, Ian J. Burgess, Sven Achenbach

**Affiliations:** 1 Department of Electrical and Computer Engineering, University of Saskatchewan, Saskatoon, SK S7N 5A9, Canada; 2 Synchrotron Laboratory for Micro and Nano Devices, Canadian Light Source Inc., Saskatoon, SK S7N 2V3, Canada; 3 Department of Chemistry, University of Saskatchewan, Saskatoon, SK S7N 5C9, Canada; 4 Mid Infrared Spectromicroscopy Facility, Canadian Light Source Inc., Saskatoon, SK S7N 2V3, Canada

**Keywords:** micro electro-mechanical systems (MEMS), Lab-on-Chip (LoC), microfabrication, polydimethylsiloxane (PMDS), UV lithography, bonding, attenuated total reflection Fourier transform infrared spectromicroscopy (ATR-FTIR), electrochemistry

## Abstract

Micro electro-mechanical systems (MEMS) combining sensing and microfluidics functionalities, as are common in Lab-on-Chip (LoC) devices, are increasingly based on polymers. Benefits of polymers include tunable material properties, the possibility of surface functionalization, compatibility with many micro and nano patterning techniques, and optical transparency. Often, additional materials, such as metals, ceramics, or silicon, are needed for functional or auxiliary purposes, e.g., as electrodes. Hybrid patterning and integration of material composites require an increasing range of fabrication approaches, which must often be newly developed or at least adapted and optimized. Here, a microfabrication process concept is developed that allows one to implement attenuated total reflection Fourier transform infrared spectroscopy (ATR-FTIR) and electrochemistry on an LoC device. It is designed to spatially resolve chemical sensitivity and selectivity, which are instrumental for the detection of chemical distributions, e.g., during on-flow chemical and biological reaction chemistry. The processing sequence involves (i) direct-write and soft-contact UV lithography in SUEX dry resist and replication in polydimethylsiloxane (PDMS) elastomers as the fluidic structure; (ii) surface functionalization of PDMS with oxygen plasma, 3-aminopropyl-triethoxysilane (APTES), and a UV-curable glue (NOA 73) for bonding the fluidic structure to the substrate; (iii) double-sided patterning of silicon nitride-coated silicon wafers serving as the ATR-FTIR-active internal reflection element (IRE) on one side and the electrode-covered substrate for microfluidics on the back side with lift-off and sputter-based patterning of gold electrodes; and (iv) a custom-designed active vacuum positioning and alignment setup. Fluidic channels of 100 μm height and 600 μm width in 5 mm thick PDMS were fabricated on 2” and 4” demonstrators. Electrochemistry on-chip functionality was demonstrated by cyclic voltammetry (CV) of redox reactions involving iron cyanides in different oxidation states. Further, ATR-FTIR measurements of laminar co-flows of H_2_O and D_2_O demonstrated the chemical mapping capabilities of the modular fabrication concept of the LoC devices.

## 1. Introduction

Miniaturized sensors are increasingly being deployed as key components of connected networks and smart systems, providing indispensable solutions to societal needs from healthcare to communications, infotainment, transportation, food processing, sustainable energy, or resource management. The worldwide demand is projected to increase to dozens of trillions by the end of the 2020s [1]. Since 1979, when Terry et al. published the first paper on a miniaturized gel chromatography system [2], micro electro-mechanical system (MEMS)-based sensors have increasingly been applied in chemical sensing, biotechnological, and life-science applications, expanding macroscopic total analysis systems (TAS) to micro TAS (μ TAS) [3], micro reactors, and eventually coining the terms *Lab-on-Chip* (LoC) and *cells on chip* systems. Such systems generally combine miniaturized fluidic handling and sensing capabilities to exploit multiple benefits when compared to classical macroscopic systems. Key benefits include increased performance per volume, portability, availability, and reliability, along with faster analysis and response times, at reduced cost and minimized fluid volumes and consumption of analytes and reagents. Ongoing improvements of the above characteristics, often combined with implementation of more complex functionalities, demand constant improvements of underlying MEMS fabrication technologies [4,5,6].

Many classical LoC-type systems are based on silicon for the availability of well-established patterning techniques, and/or on glass for its excellent chemical stability. Emerging polymer-based systems afford a range of advantages over the above-mentioned materials, including tunable materials properties (thermal, mechanical, optical, electrical) and surface functionalization. Many polymers can also directly be micro-/nanopatterned by lithographic or by mass replication techniques (e.g., embossing, molding, soft lithography approaches) as well as by precision machining and additive processes (e.g., 3D printing) [4,5,6]. Such polymer-based systems, however, generally offer limited solvent compatibility. More importantly, they must often be integrated into multi-material sandwiches or composites (e.g., with metals, ceramics, biological material) for functional or auxiliary functions of the sensing or the fluidic handling sub-units. Examples of metal thin films are seeding layers and adhesion promoters, patterned electrodes, and membranes. Polymer-based microfluidics and sensing systems are therefore usually associated with a considerable range of fabrication processes and materials, offering both opportunities for advanced sensing applications and challenges for technical implementation. Several references [7,8] exemplify typical challenges of polymer-based multi-material composites in microfluidics applications.

The present study forms part of the tremendous recent interest in microfluidic platforms for chemical and biological reaction chemistry [9]. In such systems, detection and imaging are required to spatially resolve chemical sensitivity and selectivity for the detection of chemical distributions in the device, e.g., during on-flow reactions. Sensing must therefore be rapid and non-invasive, and the devices should be reusable [10]. Besides optical methods, vibrational spectroscopy using Fourier transform infrared spectroscopy (FTIR) has emerged as a technological approach that meets those requirements [11]. Due to the strong absorption of IR radiation in aqueous media, as typically encountered in microfluidics, effective penetration lengths are limited to only a few micrometers. However, with common channel dimensions of tens to hundreds of micrometers as desirable in many applications, FTIR is hard or even prohibitive to implement, or else restricted to very shallow fluid channels, when using a standard transmission architecture where the IR radiation enters through an IR-transparent window, such as CaF_2_, crosses through the fluid channel, exits the fluid cell through a second IR-transparent window, and the remaining signal is then detected [12,13,14,15].

**Figure 1:**
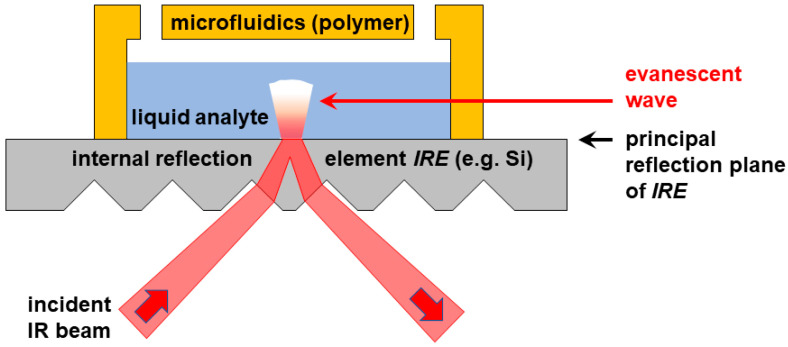
Schematic (side view, not to scale) of an ATR-FTIR configuration with microfluidics integrated onto the back side of the *internal reflection element* (*IRE*), allowing the evanescent wave to interrogate the analyte in the liquid volume at the interface to the *principal reflection plane*.

These limitations are circumvented when changing from a transmission to an attenuated total reflection (ATR) configuration as illustrated in Figure 1. In ATR-FTIR, IR radiation is shone under an oblique angle onto an optically dense material, such as silicon, which serves as an *internal reflection element* (IRE). In a prism- or grooved topography on the incident side, but a flat surface on the back plane of this IRE, radiation incident above the critical angle undergoes total internal reflection at the flat back plane (the *principal reflection plane*). As a result, a spatially confined evanescent wave originates from the principal reflection plane of the IRE. This wave can be applied to analyze matter located within about a micrometer off that plane. An emerging conceptual approach is to apply ATR-FTIR in combination with microfluidics, using the flat IRE surface as the substrate for a microfluidic cell to detect and analyze the chemicals inside the fluid channel. This technique allows one to reliably interrogate the fluid close to the interface, regardless of the overall channel height. Due to the spatial confinement and short penetration length, this approach even allows for chemical imaging [10,16,17,18].

The main aim of the research presented is to develop, verify, and apply a sequence of microfabrication processes that allows one to build *Lab-on-Chip* (LoC) devices that enable

(i) manipulation of small sample volumes of liquid analytes (e.g., co-laminar flow, mixing);
(ii) initiation and control of electro-chemical reactions on flow (e.g., using electrodes in the fluidic setup);
(iii) single-point infrared (IR) spectroscopy to detect and analyze various chemicals on-flow; and
(iv) IR imaging of areas of interest along a fluidic setup.

The fabrication processes should

(a) be repeatable and configurable;
(b) allow one to handle non-aggressive chemicals compatible with polymeric materials (e.g., in tubing, connectors, fluidic cell);
(c) allow for fluidic structures that are optically transparent (for simplified alignment to electrodes and the FTIR patterns on the back side, see Figure 1) and that can be quickly and cost-efficiently replicated, i.e., polymer-based; and
(d) allow for integration with electrochemical reaction and FTIR sensing elements (e.g., aligned and reliable bonding to/hermetic sealing with metal electrodes and silicon internal reflection elements).

Key outcomes achieved in the work presented include the following individual contributions: A microfabrication process sequence was developed that applies direct-write UV lithography to pattern a UV mask which is then used in soft-contact UV lithography to pattern a negative-tone dry resist (SUEX) as a primary microstructure which subsequently serves as a master mold for the replication of fluidic structures in a silicone elastomer, polydimethylsiloxane (PDMS). Bulk micromachining (potassium hydroxide—KOH etch) is applied to pattern the grooves into the silicon-based internal reflection elements (IREs). The back side of these IREs can be patterned with electrodes and microfluidics structures: A lift-off process is applied to pattern gold (Au) electrodes on the silicon/silicon nitride substrates, and a combination of surface activation in oxygen reactive ion etching (RIE), adhesion promotion using 3-aminopropyl-triethoxysilane (APTES), and a UV-curable glue (NOA 73) are used to ensure proper adhesion of the PDMS structure to the substrate. Alignment between electrodes and PDMS prior to bonding is achieved using a custom-designed active vacuum positioning and alignment setup. Demonstrator devices were designed and fabricated to demonstrate electrochemical reactions in the electrodes, which also ensured that nitride-coated silicon wafers sufficiently minimize leak currents otherwise observed across a bare silicon substrate. Moreover, 10 millimolar ferricyanide (potassium hexacyanoferrate (III), [Fe(CN)_6_]^3−^) and 10 millimolar ferrocyanide (potassium hexacyanoferrate (II), [Fe(CN)_6_]^4−^), both in 0.1 molar potassium perchlorate (KClO_4_), were co-flown and analyzed using the built-in electrodes for cyclic voltammograms (CVs). Single-point FTIR experiments were conducted using co-flowing water (H_2_O) and heavy water (D_2_O) to demonstrate detection of the formation of the proton/deuterium exchange product HOD.

All targeted individual goals have been reached, and approaches chosen are compatible to allow for a single integrated chip with all the properties and functionalities described above. This final step is work in progress, but has not been completed to date due to the substantial interruptions of the sudden and shocking death of the primary author, Noah Atkinson.

## 2. Materials and Methods: Micro Fabrication Process Development

Development and application of micro fabrication processes described in this paper were performed at the *Synchrotron Laboratory for Micro and Nano Devices* (*SyLMAND*) [19], Canadian Light Source (CLS), on the campus of the University of Saskatchewan, Saskatoon, Canada.

**Figure 2:**
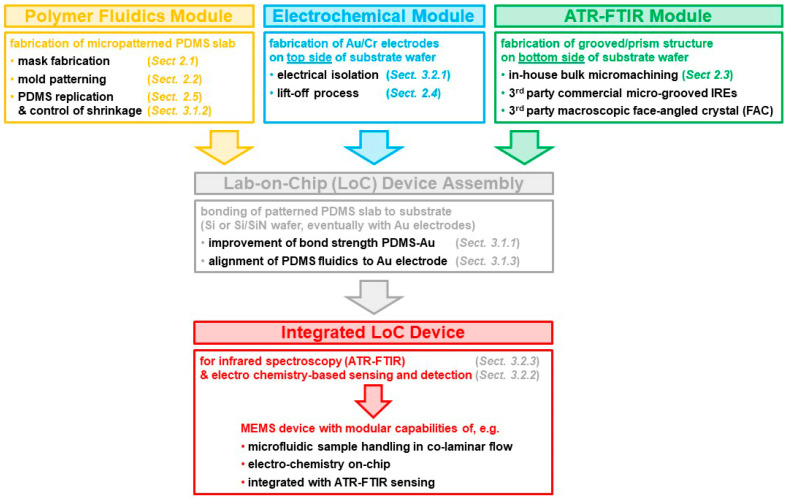
Conceptual sketch of the modular fabrication sequence for an integrated LoC device with ATR-FTIR and electrochemical functionalities. The above sketch features condensed text for increased legibility. The corresponding figure with complete text is presented in Appendix A, Figure A1, for full context and best comprehensibility.

Processes described in this section are generally adapted from the literature or our own previous research to fit the overall processing requirements of this research and to reflect equipment capabilities and restrictions at SyLMAND. While the overall processing sequence is custom designed and unique, the individual process steps described in this section generally have not been specifically optimized for this research. Figure 2 provides an overview of the individual blocks of processes. Many of them can be used modularly, e.g., to include ATR-FTIR capabilities or electrochemical functionality where needed for the specific application. Omission of any of these modules does not compromise the overall fabrication concept. Core processes are briefly described in this section for completeness.

### 2.1. Direct-Write UV Lithography and UV Mask Fabrication

The proposed integrated chip requires (i) back-side patterning of a silicon wafer with parallel grooves for ATR-FTIR analysis, (ii) front-side patterning of a metal thin film to serve as electrodes in electrochemical processes, and (iii) separate patterning of a polymer resist structure to be later used as a master mold, facilitating replication of the fluidic structures to be aligned and bonded to the silicon wafer which was previously patterned in steps (i) and (ii).

Consequently, for each chip, three independent patterning tasks were performed using ultraviolet (UV) lithography. All required chromium masks were designed and fabricated in-house. Primary patterning was achieved using a direct-write UV lithography laser writer (DWL 66+, Heidelberg Instruments, Heidelberg, Germany). The system can pattern up to 6” large substrates, providing up to 250 mW of laser power at a wavelength of 355 nm. The applied optics allow for a nominal minimum feature size of about 3 µm at a working distance of 10 mm (referred to as write mode III). Since all critical dimensions in the work reported are substantially larger, this is an adequate patterning approach. Patterning details have previously been optimized on a comparable machine [20]. The overall fabrication sequence is comparable to established Cr masking processes [4,5,6]. Briefly, commercial UV mask blanks (soda-lime glass, precoated with approximately 100 nm of chromium and 0.5 μm of an AZ-1500-series positive-tone photoresist; Nanofilm, Westlake Village, CA, USA) were exposed using the UV writer. Exposure dose values for the laser-based UV writer are not defined in standard UV lithography units (energy per area), but rather in percent of the maximum nominal laser power incident for a fixed time increment onto each pixel. These values are floating, without direct means for absolute measurements. Nominally, 80 mW was applied. Dip (puddle) development was performed in standard AZ400K developer at room temperature, for 60 s. The resulting primary resist pattern served as a processing mask during local etching of the unprotected Cr areas on the mask for 3 min in a nitric acid-based chromium etchant (Transene 1020, Transene Inc., Danvers, MA, USA). This completed defining the geometry of the Cr UV mask absorbers. In a final stripping process with acetone, isopropanol, and water, the resist etch mask was removed in acetone.

### 2.2. Soft-Contact UV Lithography for Dry Resist Mold Patterning

The final fluidic devices are replicated in PDMS using a polymer-based, 100 μm deep master mold. These molds were lithographically patterned on 4” silicon wafers. An SU-8-like, negative-tone epoxy-based resist [21] was applied to pattern the fluidic structures. However, to avoid wet chemical processing, a 100 μm thick sheet of chemically comparable dry resist (SUEX, DJ Microlaminates Inc., Sudbury, MA, USA) was attached directly to the silicon substrate. At 70 °C, the SUEX sheet was laminated [22] to the substrate, using an RSL-382S Laminator (RS Royal Sovereign, Rockleigh, NJ, USA) with fixed feed velocity. Any bubbles potentially trapped in the interface were removed using a post-lamination bake (PLB) at 85 °C for 5 min. UV exposure was performed in soft-contact shadow projection mode using the UV masks described in Section 2.1 (iii) and a mercury vapor lamp-based light house (Oriel Instruments, Stratford, CT, USA) with a low-pass filter and an exposure dose of 1000 mJ/cm^2^ at 365 nm wavelength. After a post-exposure bake (PEB) at 85 °C for 30 min in a convection oven (Binder Vacuum Drying Oven, Binder GmbH, Tuttlingen, Germany), the resist was dip-developed in propylene glycol methyl ether acetate (PGMEA) for 10 min at room temperature, followed by a rinse in isopropyl alcohol (IPA) for 5 min. Drying in the convection oven at 100 °C for 10 min was followed by a hard bake at 150 °C for 30 min.

### 2.3. Bulk Micromachining for Patterning of Grooves in the Internal Reflection Elements (IREs)

One side of the chip must be structured with grooves or a prism to allow the incident IR beam to penetrate into the silicon and reach the principal reflection plane (see Figure 1). Three options were applied and tested to obtain this topography:

Classically, a macroscopic single prism 60° face-angled crystal (FAC) made of silicon (e.g., PIKE Technologies, Madison, WI, USA) has been used. This approach offers simplicity in the overall optical setup, but it does substantially limit positions at which the fluidic setup on the opposite side can be analyzed. Furthermore, macroscopic silicon IREs may involve long beam travel through the silicon material. Associated attenuation of radiation effectively causes a cut-off of IR light at wavenumbers of approximately 1500 1/cm and less (i.e., wavelengths of approximately 6.7 μm and above). This spectral range would, however, be of substratial interest as this “fingerprint region” of the IR spectrum contains many characteristic peaks in IR spectroscopy. By reducing the size of the IRE, eventually applying micromachined IREs, the fingerprint region becomes accessible.

Secondly, such micro-grooved silicon IREs can commercially be obtained, e.g., as substrates of 9 mm by 11 mm size with 35° face angle and approximately 700 μm pitch (Universal ATR wafers, IRUBIS GmbH, München, Germany) [17]. These combine micro-scale ATR capabilities with the ability to analyze at a large variety of locations. However, geometry, angles, overall chip size, and micro structure quality are not adjustable to experimental needs.

These drawbacks can be overcome when bulk micromachining is performed in-house. Silicon wafers coated with 100 nm of silicon nitride on either side and additionally with a thin layer of AZ resist (see above) on one side are applied in this process. A UV mask as described in Section 2.1 (i) is used to pattern the resist layer, which ultimately opens up a processing window with bare silicon nitride. This is selectively etched using a Buffered Oxide Etch (BOE), a mixture of 6% hydrofluoric acid (HF) with ammonium fluoride (NH_4_F) as the buffering agent (J.T.Baker BOE 7:1, Avantor/VWR, Edmonton, AB, Canada). The etch is performed at room temperature for 4 h. After local removal of the Si_3_N_4_ thin film, the bulk of the silicon can be etched through these processing windows using potassium hydroxide (KOH) at 70 °C for about 10 h. This approach allows one to custom-design IREs to perfectly match the microfluidics on the opposite side of the substrate. First demonstrators have been successfully fabricated. However, reliable adhesion of the masking layers, simplification of the process without the need for fluorine etch chemistry, and increasing the process stability and quality are in progress (full manuscript with processing details in preparation).

### 2.4. Lift-off Process for Patterning of Gold Electrodes

After bulk micromachining one side of an internal reflection element (IRE), separately prepared polymeric microfluidics (see below) will eventually become attached to the opposite side of the IRE, i.e., to the principal reflection plane (see Figure 1). In addition to the components depicted in Figure 1, complete LoC chips as defined in Section 1 also have metal electrodes to allow for electrochemical functionality. These electrodes must be aligned relative to the microfluidic structures and are patterned onto the principal reflection plane, underneath the microfluidics.

**Figure 3:**
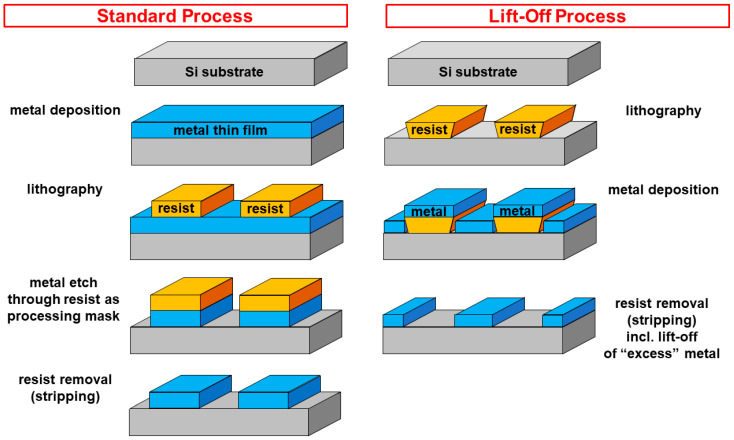
Fabrication sequence alternatives: patterning of metal thin films, such as electrodes, using a standard process (flood deposition of metal followed by lithographic patterning of resist as an etch mask to locally protect metal from removal during metal etching) or a lift-off process (lithographic patterning of resist with a back cut in the sidewalls, followed by flood deposition of metal, and final resist stripping, also lifting off unwanted metal on top of the resist).

In a standard processing sequence, the metal thin film would first be deposited onto the entire substrate, followed by lithographic patterning of resist which serves as a processing mask to locally protect the metal underneath from removal during an etch step. This concept requires good adhesion of both, resist and metal, to avoid unwanted removal of metal underneath the processing mask, or delamination of the metal thin film, during often-harsh processes required to etch the metal layer. These requirements can be eased if the alternative, a lift-off process [4,5], is applied, where the order of lithography and metal deposition steps is reversed. As depicted in Figure 3, first, a resist structure is patterned. These structures require the opposite tone relative to the standard process as they define the areas in which final metal structures, e.g., electrodes, are not supposed to be. Unlike in classical lithography, the resist sidewalls should not be as vertical as possible, but rather offer a back cut such that in the subsequent flood metallization process, only the top sides of the bare substrate and the resist are coated, but not the resist sidewalls. This enables sufficient sideways resist attack in the subsequent wet chemical resist removal (stripping) process, which also lifts off the unwanted metal parts residing on top of the resist. In this process, adhesion between resist and metal is less critical, and the metal layer itself does not need to be etched. The resist back cut can be promoted by elaborate lithographic process optimizations or by applying custom-made lift-off resists that tend to form “mushroom-like” profiles.

The electrodes applied in this research were fabricated using a lift-off process. First, hexamethyldisilazane (HMDS) was applied to the substrate as an adhesion promotor. Then, 1 mL of an MCC primer (20% HMDS, 80% PM acetate, MicroChem/Kayaku Advanced Materials, Westborough, MA, USA) was pipetted to the substrate surface, allowed to react for 10 s, and then spin coated to dry at 3000 rpm for 1 min. A dedicated negative-tone lift-off resist (ma-N 1407, micro resist technology, Berlin, Germany/Kayaku Advanced Materials, Westborough, MA, USA) was spin coated at 3000 rpm for 30 s to a nominal thickness of 0.7 μm and soft baked at 100 °C for 60 s prior to UV exposure to 350 mJ/cm^2^ at 365 nm wavelength, using the UV light house described above in soft-contact exposure mode, together with a mask fabricated in-house; see Section 2.1 (ii). Post-exposure bake (PEB) was performed at 100 °C for 10 min prior to 40 s of room temperature dip development in a tetramethylammonium hydroxide (TMAH)-based developer, ma-D 533, from the same vendor.

The patterned wafer is then flood metallized by room temperature magnetron sputtering (PVD 75 Sputtering System, Kurt J. Lesker, Concord, ON, Canada). First, an approximately 5 nm thick chromium layer serves as an adhesion promoter to silicon, followed by typically 30 nm of gold as the electrode conductor. Cr sputtering was performed for 2.5 min at 100 W with a base pressure of 3 × 10^−6^ torr and a process pressure of 5 × 10^−3^ torr Ar. Au sputtering was performed for 6 min at 90 W and the same pressure. Resist stripping and lift-off using the wet chemical remover mr-Rem from the same vendor takes approximately 1 h.

**Figure 4:**
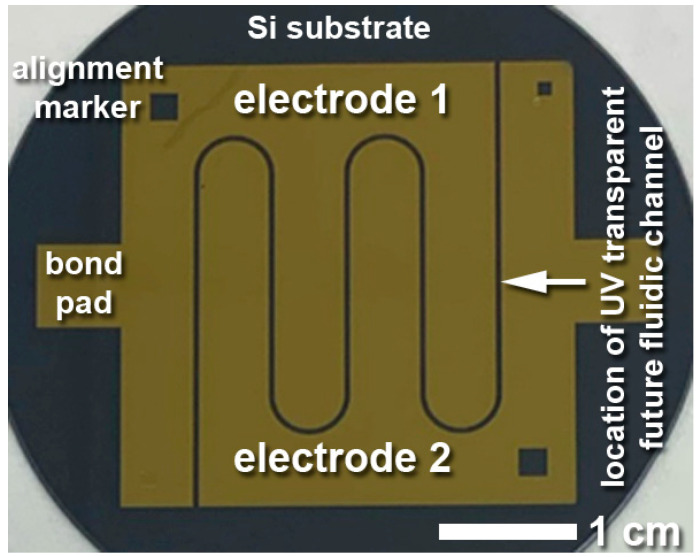
Optical image of Cr-Au electrodes patterned by a lift-off process onto a 2” silicon wafer.

Figure 4 illustrates patterned electrodes in a sample layout for snake channels on a 2” test wafer. Gold, unlike the silicon substrate, is not sufficiently IR transparent. While other electrically conductive materials, such as indium zinc oxide (IZO), are optically and IR transparent, gold has the advantage of much higher electrical conductivity, optical opaqueness facilitating optical alignment to the back side and/or the fluidics, better compatibility with established processes, and much better stability in wet chemistry and water. Gold therefore continues to be applied as the electrode material of choice, even though it fundamentally prohibits ATR-FTIR analyses. To allow for such spectroscopic measurements while still enabling electrochemical capabilities, the electrodes must be carefully designed and located. In this case, the electrodes are aligned along the future fluidic channel, always slightly (100 μm) reaching into the channel for electrical contact with the fluid, while leaving the bulk of the fluid area IR transparent. This requires alignment between the electrodes and the subsequently added fluidics layer, described in Section 3.

### 2.5. Replication of Polymer Fluid Channels into PDMS

The last individual component required for the LoC chip is the fluidic cell itself. Sidewalls and cover (lid) are replicated as a single component from the master mold described in Section 2.2, while the bottom layer is formed by the principal reflection plane of the silicon or silicon–silicon nitride IRE and eventually Cr/Au thin film electrodes. Microfabrication by replication greatly decreases fabrication timelines and costs, compared to individual lithographic processing. Microreplication is primarily conducted in polymeric materials. Most of them are also optically transparent, allowing, as an added advantage for microfluidic devices, the inspection of the mass transport inside the fluidic cell as well as optical alignment during device fabrication. Commonly used MEMS replication materials for micro hot embossing or injection molding are thermoplasts, which deliver solid and rigid micro components but require high temperature and/or pressure during fabrication, associated with expensive specialty equipment, and preferably very costly, metal-based mold masters [4]. In this work, an alternative class of polymers, elastomers, is applied in the replication process, which is soft, chemically quite resistant, transparent, and easy to work with. Polydimethylsiloxane (PDMS) is a silicone-based elastomer which is increasingly applied in fluidic MEMS for its compatibility with less-demanding molds [23] such as the SUEX-silicon-based one described above. The elastomer prepolymer is poured into the mold and cured to a soft and easily deformable structure. Besides the many advantages, using PDMS in microfluidics is also associated with a variety of technological and applications challenges: PDMS cannot be used with non-polar solvents, such as alkanes (e.g., hexane), aromatics (e.g., benzene), or acetic acid, as diffusion of the solvent into the PDMS matrix would result in massive swelling. For the applications anticipated in this research, this does not constitute a significant drawback, but extended future applications might require revisiting the fluidic material for that reason. Untreated PDMS is quite hydrophobic. Initial tests in our lab delivered contact angles with water of about 90°. This results in wetting issues with, e.g., glues applied in fluidic integration. Detailed process development to overcome this concern is described in Section 3. PDMS further noticeably shrinks upon curing, typically between 1% and 3% [24]. This constitutes a challenge when designing the proper fluidics dimensions for subsequent alignment to prepatterned layers. Considerations to overcome this challenge are discussed in Section 3 as well. Finally, the soft nature of PDMS, advantageous as it allows one to conformally follow a potentially prepatterned substrate surface, at the same time can prove to be a technical challenge when it is insufficiently rigid during alignment and tends to droop during positioning. This aspect can be optimized when adjusting the mixing ratio of base elastomer-to-curing agent (hardener), as it substantially impacts the final material stiffness.

The PDMS grade applied in this research is Sylgard 184 (Sigma-Aldrich, Oakville, ON, Canada). It was thoroughly mixed with an electric stirrer in a base elastomer-to-curing agent (hardener) ratio of 10:1. The mixture was then briefly degassed to approximately 0.1 bar to remove most of the potential bubbles. In a subsequent pouring and overcasting step, the slurry was poured into the master mold with the negative-tone layout of the 100 μm thick SUEX structures, defining channels of typically 600 μm width, along with reservoirs for entry and exit ports. The overall thickness was typically 5 mm, offering sufficient overall structural rigidity and doubling as the fluid channel surface cover as well. The samples were cured at 60 °C for 2 h (see more details and discussion in Section 3.1.2) and cooled overnight before demolding and cutting the outside walls to roughly the desired overall dimensions.

## 3. Results and Discussion

Subsequently, the individual process subgroups introduced in Section 2 are combined to form a complete fabrication sequence for a Lab-on-Chip (LoC) device. As a precondition, new process approaches and instrumentation are developed and discussed (i) to overcome the challenge of reliably bonding the PDMS fluidic slab to the substrate, (ii) to ensure minimum, or controlled, polymerization shrinkage in the PDMS slab to allow for alignment with the prepatterned metal electrodes, and (iii) to actually actively align the PDMS to the electrodes.

Finally, the LoC functionality is verified, and the capabilities are demonstrated, using the example of selected ATR-FTIR measurements and electrochemical measurements.

### 3.1. LoC Device Assembly and Fluidic Integration

#### 3.1.1. Improvement of the Bond Strength between PDMS and Gold

A primary point of potential failure in many microfluidic systems is the interface between the fluidic sidewalls and either one or two components serving as the bottom and/or lid. The components need to be reliably, and usually permanently, attached to ensure a hermetic seal. In this study, the walls and top lid are fabricated as an inseparable single slab of patterned PDMS, which needs to be bonded to the silicon-based IRE substrate (bottom). The bond must have sufficient strength to form a permanent and reliable fluidic seal and to also hold up to device handling during operation, when working with the fluidic tubing, which is connected to the PDMS slab and might exert additional forces on the bond interface. Three bond partners are to be considered, PDMS to silicon and alternatively PDMS to silicon nitride as the two IRE substrate materials used, and PDMS to the gold electrodes. The last is the dominant material combination in the test layouts. PDMS-metal bonding constitutes a substantial challenge [25], and a dedicated bond process had to be developed.

The actual bond is achieved using an adhesive dispensed onto the interface. Low-viscosity liquids are applied in order to minimize the resulting adhesive thickness and ensure good adhesive distribution. To prevent excessive further and unwanted flow on surfaces and capillary pull into the fluidic cavities, very fast curing of the adhesive once applied is ensured by ultraviolet (UV) radiation-initiated curing immediately after positioning the bond partners. Tests were performed with two UV-initiated adhesives previously successfully tested in micro- and nanofluidic sealing contexts: Dymax 191-M (Dymax, Torrington, CT, USA) has been applied in our lab for a decade to seal interfaces in nanofluidic and nanopore devices [26]. NOA 73 (Norland Adhesives, Dorset, UK) is another optically clear liquid adhesive that is UV curable and additionally partly curable and has been applied by Birarda et al. to bond advanced LoCs which require partial and full adhesive curing [27]. Overall adhesive performance in the present study was comparable. Dymax 191-M has limited capability to withstand solvents [28], including acetic acid and hexanes. As these are solvents that are also incompatible with PDMS, this property is not considered a disadvantage in this study. NOA 73, however, offers the additional advantage of partial curability, i.e., control over viscosity, that could be exploited in an optimized delivery approach (see below). NOA 73 was therefore applied in the bonding process described here.

The hydrophobic properties of PDMS (see Section 2.5) cause the adhesive to form beads and a non-uniform layer, which is prohibitive for successful bonding. After patterning, and prior to dispensing the adhesive, the PDMS bonding surface therefore requires surface functionalization.

In a first functionalization step, PDMS was activated with oxygen plasma in a reactive ion etcher (RIE; Plasmionique FLRIE-300C, Varennes, Québec). Using a pure oxygen (O_2_) environment of about 0.2 mbar pressure, a flow rate of 50 sccm, and a plasma power of 50 W for 60 s, chemically active, dangling hydroxyl groups (–OH) were created on the PDMS surface. This activation by itself is insufficient to deliver satisfying bond results with the NOA 73 adhesive and the gold electrodes. However, Agostini et al. [25] proposed 3-aminopropyl-triethoxysilane (APTES) for successful functionalization of PDMS, allowing for covalent bonding. Immediately after activation, PDMS was therefore dipped into 1% APTES (Sigma-Aldrich, Oakville, ON, Canada) for 20 min and subsequently dried.

After this two-step functionalization, the modified PDMS surface was coated with NOA 73 adhesive and placed onto the IRE substrate with gold electrodes. NOA 73 requires 4 J/cm^2^ for full curing [29]. Absorption of UV radiation in the PDMS slab prior to reaching the bonding interface increases the exposure time with 365 nm radiation substantially. An exposure time of up to 1.5 h was reported previously [27]. In the configuration studied here, 10 min of exposure at approximately 20 mW/cm^2^, i.e., 12 J/cm^2^ incident onto the PDMS slab, was sufficient to cure the adhesive. Parameter variations with exposure times of up to 2 h did not result in better adhesion. In all cases, the adhesion between PDMS and gold exceeded that of the sputtered Cr/Au thin film on the silicon IRE. When forced apart, gold would adhere to PDMS instead of the silicon wafer. Process variations were also conducted, resulting in the necessity of both functionalization steps and the adhesive application and curing steps to allow for robust and reliable bonding. Omission of any of the mentioned steps annihilates the bond success.

A small fraction of the adhesive entered the fluid channels. To minimize this effect, various delivery approaches for the adhesive have been contemplated and tested: For the original process development on un-patterned blocks of PDMS, spin coating NOA 73 at 3000 rpm for 60 s was successfully applied. Using this approach on patterned PDMS structures would obviously result in micro channels being flooded with the adhesive. As an improvement, a stamping method was developed and tested. In this process variation, the adhesive was spin coated with the same parameters to a sacrificial silicon wafer, from where the adhesive was transferred to the PDMS walls in a soft lithography-like procedure [5] which involves dipping the PDMS slab onto the coated sacrificial wafer. Theoretically, in this way, only the topographically prominent areas of the PDMS are coated, and not the recessed fluidic channels. Practically, this benefit proved to be largely lost if detaching the PDMS from the wafer included any sideways sliding motion. Careful manipulation following this alternative process has been applied in the demonstrators described below. Ongoing research investigates further optimizations of this process variation, exploiting NOA 73’s ability to be partially cured. In this scenario, the adhesive is slightly pre-cured on the sacrificial wafer, increasing the viscosity and decreasing the risk of sideways sliding when detaching the PDMS slab. Alternatively, better control of the amount of adhesive dispensed could potentially be obtained using a roll-on approach from a cell foam reservoir.

**Figure 5:**
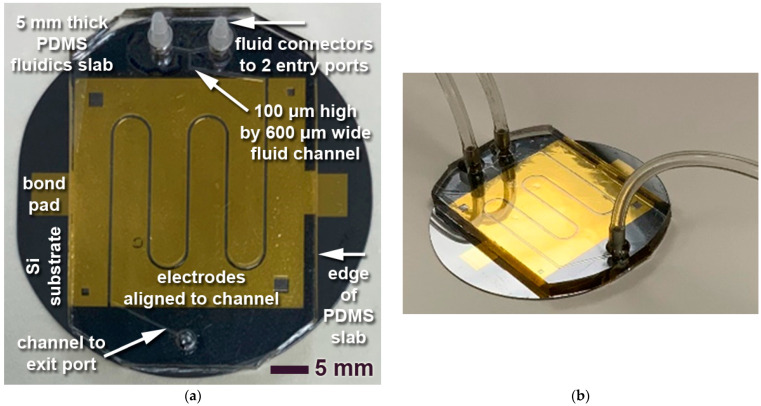
Optical image of an LoC chip with a 5 mm thick PDMS slab aligned and bonded to a 2” silicon substrate patterned with Cr-Au-electrodes (see Figure 4). The bottom side of the PDMS slab contains a snake micro mixer (channel dimensions: 100 μm high by 600 μm wide). (**a**) Top view; (**b**) Arial view with tubing attached to the 3 ports.

Figure 5 shows a top view (panel a) and arial view (panel b) of a test sample with a 5 mm thick patterned PDMS slab successfully bonded to a 2” silicon wafer with patterned gold electrodes.

#### 3.1.2. Control of PDMS Polymerization Shrinkage

Polymerization of many polymers applied in MEMS technologies is associated with shrinkage of typically between a few % and up to 20% [4]. PDMS shrinkage during the curing process typically amounts to 1% to 3% [24]. When fabricating both the prepatterned electrodes on the IRE and the PDMS slab to nominal dimensions, shrinkage in the PDMS of up to 5% was observed. This has a substantial impact on the device functionality. The shortest distance between neighboring alignment markers as depicted in Figure 4 and Figure 5 is about 27 mm, which means that their separation can shrink up to 1.3 mm in the PDMS slab relative to the substrate dimensions. This is far larger than the markers themselves and renders proper alignment practically impossible. More importantly, though, this deviation greatly exceeds the alignment needs, as it is more than twice the channel width and more than three times the gap between the electrodes. Properly overlaying the electrodes and their gaps with the fluid channel therefore becomes impossible.

If the PDMS shrinkage was isotropic and repeatable, the design of the PDMS structures could be adjusted for the subsequent shrinkage. That is, a modified layout could compensate for a precisely known amount of shrinkage, even if this amount is comparably large.

In a more fundamental approach, as chosen here, the shrinkage itself could be minimized. According to [24], decreasing the curing temperature from, e.g., 120 °C to room temperature and increasing the base elastomer-to-curing agent (harder) ratio from 10:1 to 20:1 can substantially reduce the observed shrinkage by up to one order of magnitude. Changing the composition has a lower impact and also adversely impacts the mechanical properties of the cured PDMS. Therefore, in the study presented, only the curing conditions were adjusted from initially 60 °C for 2 h to room temperature curing at 21 °C overnight. This resulted in a shrinkage of much less than 1%, typically around 0.3%, which was acceptable for most layouts tested. More advanced layouts might require additional adjustment of the layout dimensions, as explained above.

#### 3.1.3. PDMS-Electrode Alignment Control with Vacuum Positioning and Alignment Setup

As explained in Section 2.4, gold was chosen as the preferred electrode material. Since gold is not transparent in the IR spectral range, the electrodes may not obstruct the path of IR radiation in ATR-FTIR measurements. The electrodes must, however, reach slightly into the fluidic channel to ensure electrical contact with the fluid to enable electrochemical processes. For the demonstrators in this study, the electrodes protrude 100 μm into the 600 μm wide fluidic channel from either side, leaving a 400 μm wide IR-transparent gap for FTIR experiments. The patterned PDMS slab must therefore be well aligned relative to the electrode/IRE substrate sandwich.

**Figure 6:**
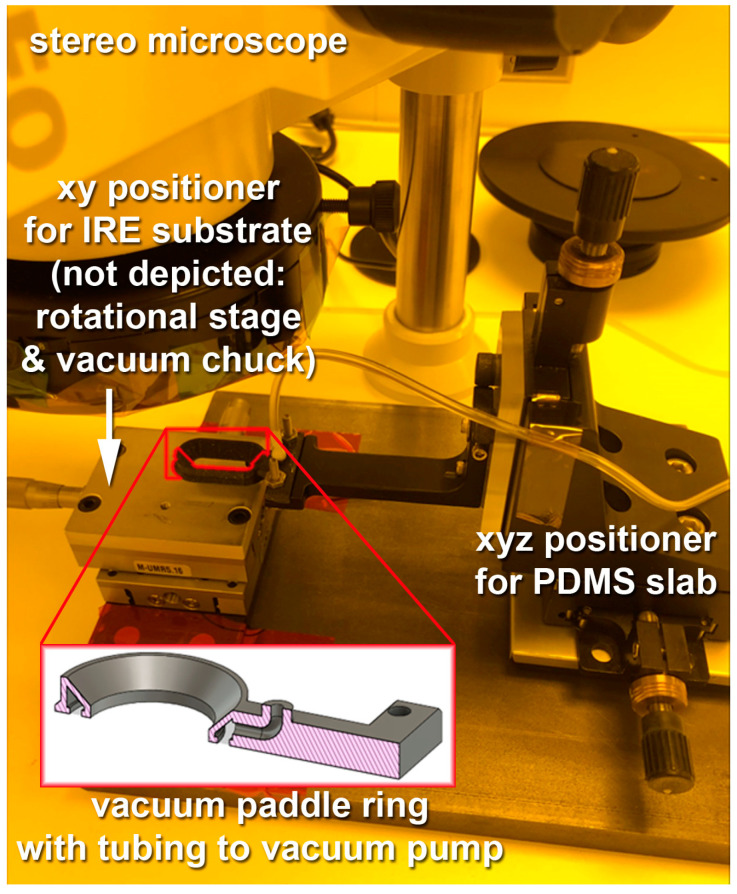
Vacuum positioning and alignment setup with vacuum paddle ring mounted to a 3-axes positioner on the right side and connected to a vacuum pump. Substrate positioning system on the left side (rotational stage, vacuum chuck, and sample itself not shown). The setup is located underneath a stereo microscope, allowing one to observe alignment markers on the substrate and in the PDMS slab top-down through the central opening in the vacuum paddle ring.

An alignment and inspection setup was developed and custom built for this purpose. It takes advantage of different properties of the PDMS polymer, i.e., its light weight, satisfying mechanical rigidity, smooth surface, and optical transparency. The key component of the alignment instrumentation is a *vacuum paddle ring* custom designed and 3D printed from polylactic acid (PLA). A variety of such paddles was fabricated to accommodate a range of shapes and sizes of PDMS slabs. The vacuum paddle ring is mounted to a three-axes positioner (x,y,z) with manual operation, 10 μm precision, and 25 mm travel range in any axis. The paddle is connected via tubing to a vacuum pump, as illustrated in Figure 6. The inset shows that the paddle has a uniform vacuum cavity at the bottom.

The PDMS slab, which was previously activated and coated with adhesive on its bottom side as described in Section 2, is positioned centrally below the vacuum paddle ring and brought into contact. This instantaneously creates a vacuum seal between the bottom side of the paddle and the top side of the PDMS slab, allowing the z positioner to lift the paddle together with the PDMS slab.

The IRE substrate with the patterned gold electrode is mounted onto a second three-axes positioner (x, y, and rotation) with vacuum chuck on top. All linear stages were pre-existing in the lab, while the rotational stage was specifically acquired (PR01/M, ThorLabs, Newton, NJ, USA) along with a large aluminum breadboard to firmly mount all components (MB306/M, ThorLabs, Newton, NJ, USA). The rotational stage and vacuum chuck are not depicted on the left side of Figure 6. The vacuum chuck holds the substrate firmly in place.

The entire setup is located underneath a stereo microscope (Zeiss SteREO Discovery V8, Carl Zeiss Canada, Toronto, ON, Canada). Thus, the alignment markers that were patterned into the gold electrodes on the substrate and into the fluidic layout in the PDMS slab can be observed top-down through the central opening in the vacuum paddle ring, and relative positioning between the paddle with the PDMS and the substrate can be performed in all three spatial directions as well as rotationally, until the best possible alignment is observed. At this point, the PDMS is lowered to make contact with the substrate. The vacuum is switched off, venting the vacuum paddle ring and releasing the PDMS slab for subsequent curing of the adhesive as described in Section 2.

The PDMS slab must be sufficiently rigid not to droop on the outer edges, potentially breaking the vacuum seal prematurely. The vacuum paddle ring should also be positioned relatively symmetrically with respect to the PDMS slab to avoid excess asymmetric gravitational forces. This is why a range of different paddle geometries were designed, fabricated, and tested. Moreover, changing the PDMS composition would influence the mechanical properties and could have an adverse impact on the alignment process. The weight-lifting capacity of the vacuum paddle ring is a function of the vacuum seal surface area. At close to 1 bar differential pressure, the designed paddles have at least 180 mN (close to 20 g) of carrying capacity, which far exceeds the weight of the PDMS slabs in this study.

**Figure 7:**
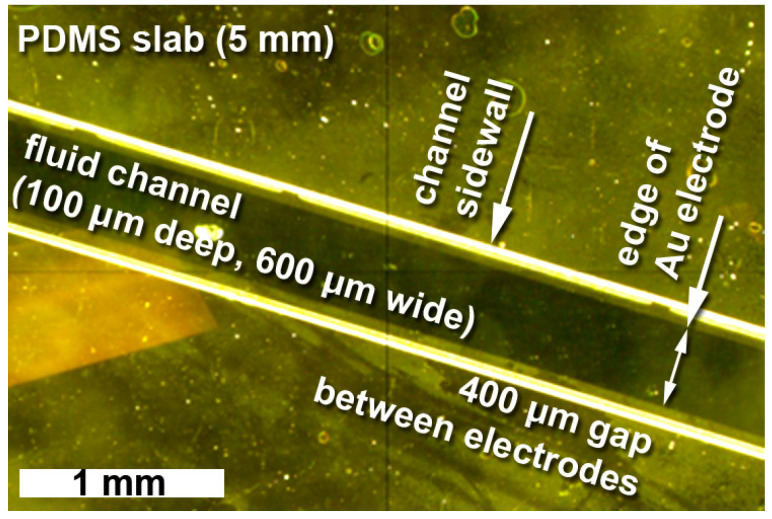
Stereo microscopical image illustrating the alignment quality evident from the symmetrical protrusion of the gold electrodes relative to the fluid channel in the PDMS slab.

The active alignment approach described has reliably delivered good alignment results. For example, the stereo microscopical image in Figure 7 shows a top view of a patterned PDMS slab bonded to gold electrodes. The sidewalls of the 100 μm deep and 600 μm wide channel appear as bright lines due to some scattered light. The electrodes protrude 100 μm on either side into the channel, leaving a gap for FTIR measurements of 400 μm. The almost-perfect symmetry indicates the high alignment quality.

#### 3.1.4. Fluidic Integration

The test layouts with 600 μm wide fluidic channels have the channels widened to a 3.5 mm diameter circular reservoir at all entry and exit port locations. This gives sufficient tolerance to allow for manually punching a medical tissue sample stamp (needle) through the top of the PDMS slab, creating pores that connect to the fluidic channel at the bottom of the PDMS slab. Barbed fittings (Super-Flow Barbed Fitting, 1/16” inner diameter, polyethylene; McMaster-Carr, Los Angeles, CA, USA) were pressure fit into these pores and glued with UV curable glue at the top to form a fluidically tight and mechanically robust seal. PTFE tubing with 1/16” outer diameter and 0.75 mm inner diameter (Chromatographic Specialties Inc., Brockville, ON, Canada) was connected to the barbed fittings. The left panel of Figure 5 shows two entry port barbed fittings at the top of the image, while the right panel in Figure 5 shows three tubes connected to all fluidic ports.

Pumping through the LoC chips was achieved using gas-tight glass syringes (model 1001 TLL, PTFE Luer Lock, 1 ml, 4.61 mm inner diameter, Hamilton Company, Reno, NV, USA) and one syringe pump (NE1000, New Era Pump Systems Inc., Farmingdale, NY, USA) for each syringe, allowing for co-flowing fluids.

### 3.2. LoC Device Functional Verification

Based on the process development and system integration described above, LoC chip demonstrators were designed and fabricated to verify the intended device functionality.

#### 3.2.1. Electrochemical Reactions Experiments—Electrical Isolation

First, electrical contacting of the LoC chip was performed without a fluid, i.e., leaving an air gap between the electrodes. The resistance between the electrodes was measured to be in the order of 10 kΩ, consistent with textbook silicon resistivity values and the sample geometry. This means that the conductivity of the silicon substrate was too high to perform meaningful electrochemical experiments, which require good electrical isolation between the electrodes in the absence of a liquid serving as an electrolyte. Since the ATR-FTIR approach depends on silicon, changing from semiconducting silicon to an electrical insulator, such as glass, as the substrate was not a long-term option. We still verified the fabrication process compatibility on glass for potential future use. As a viable approach for the current LoC device, the silicon substrate and the Cr/Au electrodes required an additional thin film insulator in between. Silicon nitride (Si_3_N_4_) is a good electrical insulator, partially IR transparent, and commonly available as a thin film coating on silicon wafers. Device fabrication according to the processes described in this section and Section 2 and electrical isolation on silicon wafers coated with 100 nm of nitride on either side (University Wafer, Boston, MA, USA) was successfully tested.

**Figure 8:**
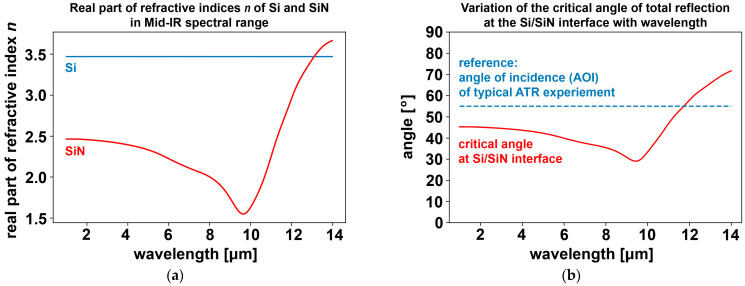
Impact of a silicon nitride (SiN) thin film coating on a silicon (Si) substrate as a function of wavelength in the mid-IR spectral range. (**a**) Refractive indices *n* of Si (blue) and SiN (red); (**b**) Variation of the critical angle of total reflection at the Si/SiN interface. The angle of incidence (AOI) of typical ATR-FTIR experimental setups using micro-grooved IREs (55°) is plotted as a dashed reference line. The critical angle varies significantly over the mid-IR spectrum and exceeds 55° only at long wavelengths.

Adding 100 nm of silicon nitride (SiN) to the substrate wafer ensures electrical isolation between the electrodes as required for electrochemistry. However, the added layer has a potential impact on the FTIR performance. At wavenumbers of 2000 1/cm and above, corresponding to wavelengths of 5 μm and below, SiN offers excellent IR transparency, while the transmittance of 100 nm SiN reduces to about 83% at wavelengths around 11 μm, as evident from Figure S1 in the Supplementary Materials, which was based on refractive index data from [30,31] and calculations using an open access tool [32]. This reduction of up to 17% can be considered a very moderate attenuation of the expected signal. It does, however, coincide with a spectral area of prime interest in vibrational spectroscopy, as a lot of molecular structural information can be obtained in the wavenumber range of approximately 400 to 1600 1/cm (corresponding to wavelengths of 25 μm to 6.25 μm). Removal of the SiN coat between the gold electrodes would therefore at least be potentially beneficial. More critically, though, the added SiN layer impacts the critical angle of total reflection in the silicon internal reflection element (IRE). Figure 8, panel (a), compares the real parts of the refractive indices of Si and SiN in the mid-IR spectral range. While the refractive index for silicon is almost constant at approximately 3.45, the refractive index for silicon nitride varies by more than a factor of 2 within the spectral range of interests from 2 μm to 14 μm. This has an effect on the propagation of the IR radiation, as illustrated in Figure 8, panel (b). For a pure silicon-based IRE, the angle of incidence (AOI, between incident IR beam and the direction orthogonal to the substrate surface) is typically at 55° (dashed reference line in panel (b) of Figure 8), which is above the critical angle for total reflection in Si, the precondition for a spatially constrained evanescent wave to emerge from that surface. For a Si/SiN interface, and the AOI still at 55° as fabricated and as embedded in common ATR-FTIR setups, this AOI is above the critical angle of total reflection for that internal Si/SiN interface for most of the spectral range of interest, up to about 13 μm. In this spectral range, an evanescent wave will therefore be emitted at that internal interface, rather than transmitting the IR light to the top of the SiN layer. While some of the evanescent wave will still penetrate the 100 nm thin SiN coat to then interrogate the liquid above, better ATR-FTIR performance is expected if the SiN thin film between the electrodes is selectively and locally removed. This is easily implemented using hydrofluoric acid (HF) as an etchant, with the gold electrodes doubling as the etch mask to protect the SiN layer as the electrically isolating layer underneath.

On the opposite side of the substrate, where the silicon substrate contains the groove microstructure, the presence of a surface coat of SiN is irrelevant. This is because the top SiN coat is removed during bulk silicon etching from optically active grooved areas anyway, while IR radiation incident to the ridges (plateaus) between the grooves, whether coated with SiN or not, does not reach the detector and therefore carries no analytical signal [33].

#### 3.2.2. Electrochemical Reactions Experiments—Cyclic Voltammetry

*Cyclic voltammetry* (CV) was performed as a proof-of-concept experiment. CV is a common electroanalytical technique applied to study reversible redox processes of analytes of interest dissolved in an electrolyte, the stability of reaction products, or the presence of intermediates [34]. In a cyclic sweep, the working electrode potential in the three-electrode potentiostat is first increased. In the presence of reducible analytes in solution, electrical reduction will create a cathodic current, measured between the working and counter electrodes. As the concentration of reducible analyte is reduced, the cathodic current decreases. In a cyclic sweep, the driving working electrode potential is then reversed, resulting in analyte re-oxidation and the appearance of an anodic current. For fast electron transfer in stationary (non-agitated, stagnant) systems, the current is limited by diffusion, and the CV plot of current-versus-potential (voltammogram) displays characteristic peaks and waves [35].

In initial CV experiments, redox reactions between inorganic complexes of iron in different oxidation states were measured, according to Equation (1):

[Fe^(III)^(CN)_6_]^3−^ + e^−^ ↔ [Fe^(II)^(CN)_6_]^4−^ (1)

**Figure 9:**
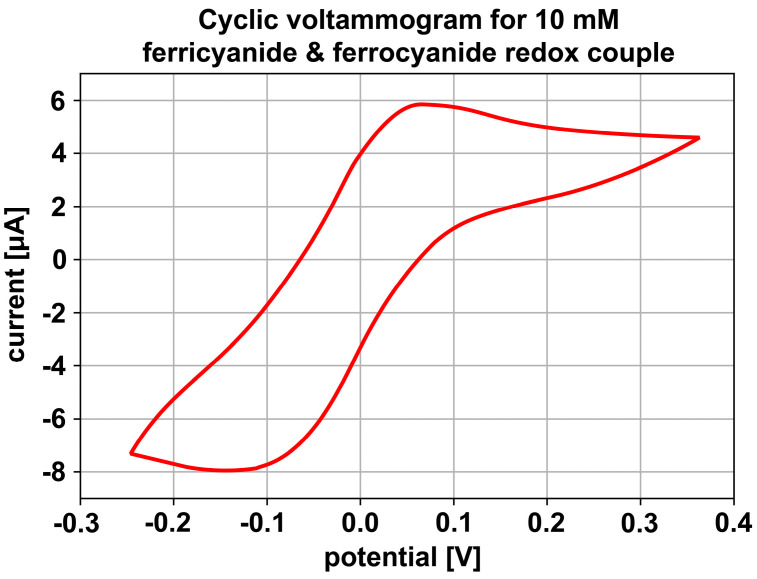
Electrochemistry proof-of-concept measurement with the LoC device. Cyclic voltammogram for ferricyanide and ferrocyanide redox couple (10 mM) in potassium perchlorate (100 mM) and a sweep rate of 50 mV/s.

First, 10 millimolar ferricyanide [Fe^(III)^(CN)_6_]^3−^ and 10 millimolar ferrocyanide [Fe^(II)^(CN)_6_]^4−^, both in 0.1 molar potassium perchlorate (KClO_4_), were inserted through two separate inlet ports and initially co-flown. To obtain stagnant conditions for simple, textbook-like voltammograms, the flow was then stopped and CV was performed under quiescent conditions, at a sweep rate of 50 mV/s. One of the LoC Cr/Au electrodes was connected as the working electrode, while counter electrode and reference electrode were shorted together on the second LoC electrode. The solution was purged with inert gas (Argon) to remove dissolved oxygen before injection into the device. The electrode was cycled for approximately 1 h in electrolyte to clean it, with a stable CV indicating that cleaning was complete. After injection of ferri/ferrocyanide, the initial cycles still showed some variation from cycle to cycle, indicating that the gold electrodes were still slightly contaminated from the fabrication process and cleaned by the first CV cycles. After about six cycles, the voltammograms obtained were identical from cycle to cycle. Figure 9 illustrates such a voltammogram under stationary conditions.

The CV shown in Figure 9 shows clear evidence of oxidation and reduction of the redox-active iron species in solution. Anodic and cathodic current peaks are observed at roughly +0.075 V and −0.125 V, respectively, with diffusional tailing consistent with mass-transport limited processes. The peak-to-peak separation between cathodic and anodic peaks is approximately 200 mV, much greater than the theoretically expected 59 mV for a one-electron process at 25 °C, such as that studied here [35]. This, and the distorted shape of the CV overall, is likely the result of hindered mass transport in the microfluidic channel due to the complex geometry of the electrodes. Nevertheless, the CV results prove the ability of the LoC device concept and fabrication sequence to enable electrochemistry on-chip.

#### 3.2.3. Single-Point FTIR Experiments

Attenuated total reflection Fourier transform infrared spectromicroscopy (ATR-FTIR) single-point experiments were conducted to demonstrate the infrared sensing capabilities of the fabrication process module (see Figure 2). For the purpose of demonstration, no electrochemical capabilities were required from the demonstrator, such that the electrochemical module was not integrated into the specific device, i.e., electrodes and the additional approach to ensure adhesion between PDMS and gold could be omitted. The channel width in this snake channel layout was 500 μm. For the integration of the topography on the internal reflection element (IRE), a macroscopic face-angled crystal was applied (see Section 2.3). The measurements were performed at the *Mid-Infrared Spectromicroscopy* beamline, Canadian Light Source (CLS) [36,37]. The beamline is equipped with a custom-designed horizontal microscope endstation (hATR, [16,17]) and a focal plane detector with 64 by 64 pixels. Customized optics allow for 3× magnification. The beamline supports the use of different sources of radiation, either synchrotron-based infrared radiation or radiation from a globar. For the measurements performed, the globar source was applied, and single-beam measurements of 128 co-added interferograms with 16 1/cm resolution were recorded for reference and sample measurements. A full account of the measurement setup and the data obtained is given in a dedicated paper recently published by our team [10].

**Figure 10:**
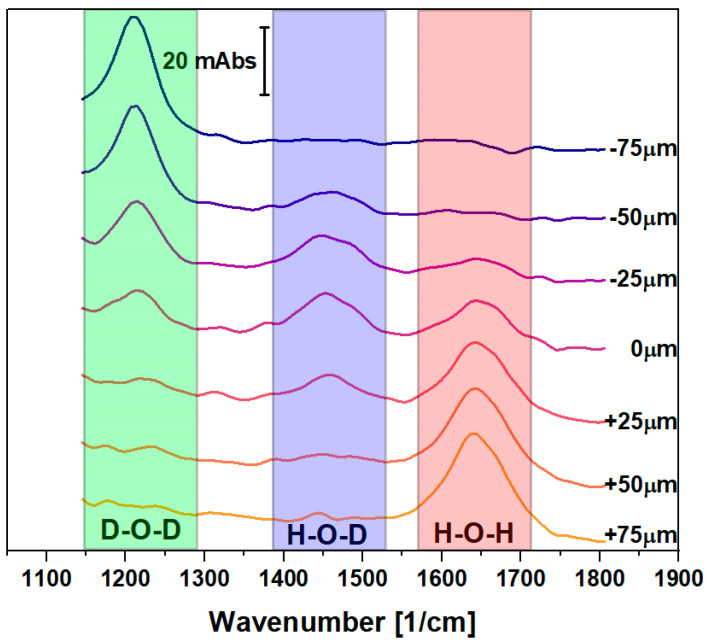
Absorbance spectra for D_2_O and H_2_O as well as for the proton/deuterium exchange product HOD, recorded in an FTIR demonstrator. The various curves represent selected sideways positions across the 500 μm wide PDMS channel. D_2_O is observed in the left half (upper curves), H_2_O on the right side (lower curves), and HOD in the center. The spectra are taken of the solution near the middle of the third channel, a total distance of 3 cm from the point at which the two fluids intersect.

Water (H_2_O) and heavy water (D_2_O) were pumped through the two entry ports of the PDMS snake channel layout and co-flown, at a rate of 1.8 ml/h, eventually leading to the formation of the proton/deuterium exchange product HOD. Absorbance spectra of H_2_O are dominated by a low-frequency bending mode peak around 1640 1/cm wavenumbers and a higher-intensity O–H-stretching mode peak around 3350 1/cm, while absorbance spectra of D_2_O show respective peaks around 1205 1/cm and 2485 1/cm. The exchange product HOD shows both peaks in a less intense way. Only HOD, though, has an additional characteristic bending mode peak around 1455 1/cm. Representative absorbance spectra were obtained with the demonstrator device measured in the third channel, near the center of the device. This corresponds to 3 cm downstream of the point of intersection of the two reagents. Figure 10 illustrates the absorbance as a function of the wavenumber. The low frequency bending mode spectral windows are highlighted as a green box for D_2_O (D–O–D) at 1205 1/cm, blue for H–O–D at 1455 1/cm, and red for H_2_O (H–O–H) at 1640 1/cm. The orientation of the fluidic channel is orthogonal to the prism on the bottom side, allowing the IR beam to scan across the channel width and taking spectra at various positions across the 500 μm wide channel. In Figure 10, seven individual spectra are represented for seven sideways positions across the middle of channel, each separated by 25 μm, for one fixed downstream position. This middle position represents the diffusion zone in the experiment and contains the most important data. The curves are overlaid and offset on the y-axis, with the y-axis scale given in the inset. The spectra indicate that heavy water (D_2_O) is flown through the left side of the channel (top four curves), at channel positions from the left channel wall at −250 μm, with the first spectrum being represented for a position of −75 μm, to the center at 0 μm, while the other half of the channel does not show peaks in the corresponding spectral range. By contrast, water (H_2_O) is present primarily in the bottom curves, i.e., the right side of the channel, at positions from 0 μm to 250 μm (of which positions up to 75 μm are represented in the graph). A small H–O–D peak is observed right in the center of the channel, around 0 μm, corresponding to the center curves in Figure 10.

**Figure 11:**
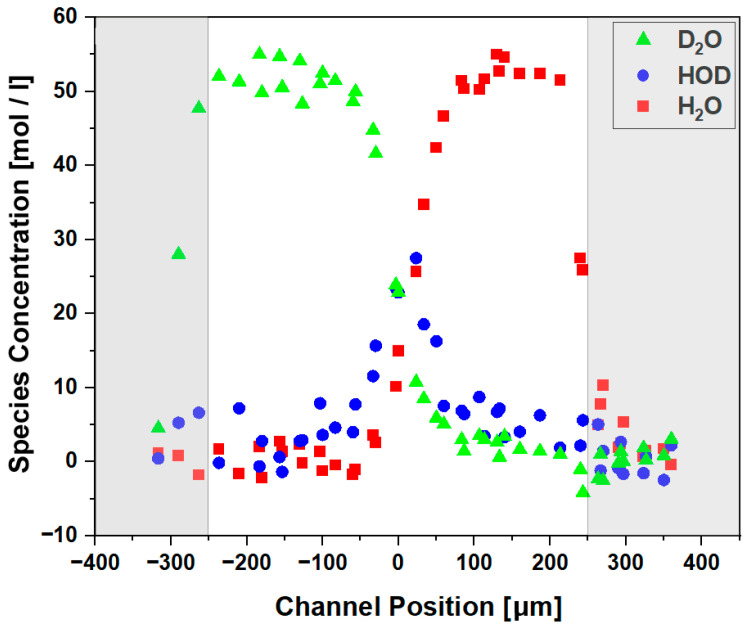
Co-flowing diffusion profiles across a 500 μm wide channel for one fixed downstream position, indicating relative contributions to the overall flow. Maximum values for D_2_O on the left side (green triangles), H_2_O on the right side (red squares), and HOD contributions only in the center (blue circles).

The data can be evaluated to create co-flow diffusion profiles. Figure 11 exemplifies this by illustrating concentration profiles of species across the channel. Using calibration curves for D_2_O, HOD, and H_2_O as further described in ref. [10], the respective absorbance areas were converted into concentrations. D_2_O predominates on the left side (green triangles), H_2_O dominates on the right side (red squares), while HOD contributions are present only in the center (blue circles). The gray sidebars indicate the edge of the fluidic channel, where, accordingly, the fluid concentration approaches zero.

With the fabrication process capabilities demonstrated for all modules described above (see Figure 2), i.e., the polymer fluidics module, the electrochemical module, and the ATR-FTIR module, current work in our lab involves applying all three modules to prepare a fully integrated device with electrochemical reaction triggering and ATR-FTIR imaging and spectroscopy capabilities. This work was interrupted by the untimely passing of the first author of this manuscript.

## 4. Conclusions

Polymer-based Lab-on-Chip (LoC) devices often comprise micro- or nanopatterned multi-material composites, offering opportunities for advanced sensing applications due to the wide selection of materials. However, these devices also impose challenges to develop and optimize a growing range of microfabrication processes for technical implementation. This manuscript describes the process development to microfabricate LoC devices applicable in microfluidics combined with infrared vibrational spectroscopy and microscopy as well as in electrochemistry. Successful application of demonstrator devices in attenuated total reflection Fourier transform infrared spectroscopy (ATR-FTIR) and electrochemistry-based sensing and detection is demonstrated.

Polymers, silicon, and metals are patterned in a modular fabrication sequence. Patterning of the fluidic structures is based on ultraviolet (UV) lithography and replication. Direct-write laser lithography is applied to pattern UV masks subsequently applied in soft-contact UV lithography in SUEX dry resist to create a master mold on a silicon substrate. This tool then facilitates replication in polydimethylsiloxane (PDMS) elastomers as the final fluidic structure. Fluidic channels of 100 μm height and 600 μm width in 5 mm thick PDMS were fabricated on 2” and 4” demonstrators.

A silicon wafer, for some demonstrators in this study coated with 100 nm silicon nitride, doubles as an ATR-FTIR-active internal reflection element (IRE) patterned with grooves or prisms on one side, and as a flat substrate for the LoC fluidics on the opposite side. In one process variation, the Si wafer is patterned by bulk micromachining in-house, while in alternative variations, externally purchased micro-grooved wafers or macroscopic face-angled silicon crystals (FACs) can be applied. The opposite, flat surface of the silicon substrate serves as the principal reflection plane in ATR-FTIR, emitting the evanescent wave applied to interrogate the analytes in solution close to the interface between silicon substrate and PDMS fluidics. For electrochemical functionality, sputtered thin film electrodes of 5 nm chromium and 30 nm gold are structured by a lift-off process on the un-patterned flat side of the substrate. For sufficient electrical isolation between the electrodes in electrochemical experiments, the conductivity of the silicon surface must be reduced, which was achieved using a silicon wafer coated with 100 nm silicon nitride (SiN) prior to electrode patterning. Between the electrodes, the SiN thin film is later selectively and locally removed by hydrofluoric acid (HF) to improve ATR-FTIR performance.

Finally, the patterned PDMS slab is bonded to the wafer/gold electrode sandwich. Reliable adhesion between these bonding partners constitutes a challenge, which has been overcome by a combination of PDMS surface activation with oxygen plasma in a reactive ion etcher, immediately followed by functionalization with 3-aminopropyl-triethoxysilane (APTES). Resilient bonding of this primed surface to the substrate wafer is achieved using a UV-curable glue (NOA 73).

On-flow chemical and biological reaction chemistry is compatible with the proposed fabrication concept. In this case, the gold electrodes must be in contact with the fluid of interest within the fluidic cell. The electrodes are, however, opaque in the infrared wavelength range and could therefore prohibit simultaneous electrochemical and ATR-FTIR measurements. Precisely limiting the protrusion of the electrodes into the channel while ensuring that the bulk of the channel is free of gold is a conceptual precondition. This requires adjusted layouts of the fluidics and electrode layers, as well as proper alignment between them. A custom-designed active vacuum positioning and alignment setup was developed. It incorporates manual multi-axes positioners, a stereo microscope, and a custom-design 3D-printed vacuum paddle ring. This allows one to apply a vacuum seal between the paddle and the PDMS slab for controlled, precise positioning relative to the prepatterned substrate. After this alignment, the bond partners are brought into contact by lowering the PDMS slab onto the substrate, and a permanent bond is created by UV curing of the adhesive. Alignment results obtained prove the concept.

The demonstrated fabrication sequence is modular. For instance, patterning of micro-grooved silicon IREs can be part of the described process or be performed externally. Likewise, electrodes for electrochemical functionality can be added if needed for the application. The fabrication sequence allows one to implement all these aspects, or just those required for the specific sensing task. Demonstrators fabricated for this manuscript to prove the fabrication capability and measurement suitability concepts incorporated the functionality required for the individual devices. We are currently working on a completely integrated device.

Electrochemistry on-chip functionality was demonstrated by cyclic voltammetry (CV) of redox reactions involving a commonly studied redox couple, ferri/ferrocyandide, in different oxidation states. Further, ATR-FTIR measurements of laminar co-flows of H_2_O and D_2_O established the local concentration distribution of each species along with the formation of the proton/deuterium exchange product HOD. This demonstrated the FTIR capabilities of the microfabrication concept.

## Figures and Tables

**Figure A1:**
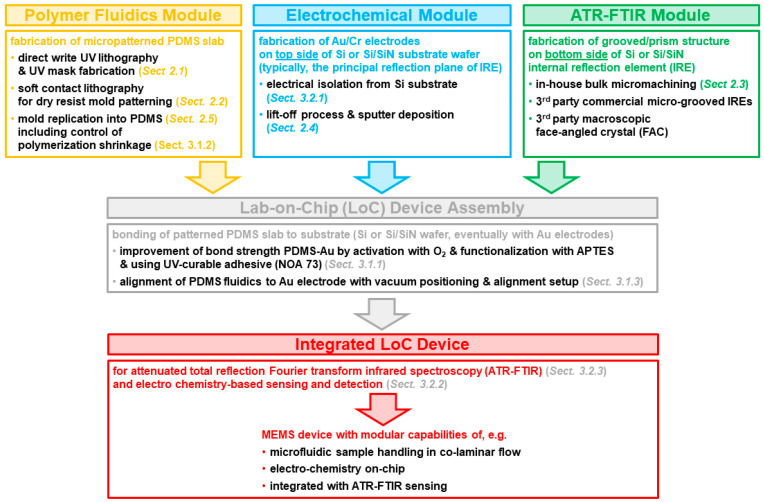
Conceptual sketch of the modular fabrication sequence for an integrated LoC device with ATR-FTIR and electrochemical functionalities.

## Data Availability

The complete list of optimized/final process parameters and details on the infrastructure used has been provided in the manuscript to allow for reproduction of the research presented. Selected process parameters that did not prove to be successful have also been discussed in the manuscript where this data could be of general interest. The majority of the research data supporting the reported results has therefore been given in the manuscript. Further measurement details were compiled from various notebooks and lab books of Noah Atkinson. Due to his unexpected death, the handwritten data are not yet available in a publicly presentable form.

## Supplementary Materials

The following supporting information can be downloaded at: https://www.mdpi.com/article/10.3390/s23146251/s1, Figure S1: Transmission of 100 nm of silicon nitride.

## Appendix A

Figure A1 below is a conceptual sketch of the modular fabrication sequence for an integrated LoC device with ATR-FTIR and electrochemical functionalities as presented in Section 2. The figure was optimized for full context and best comprehensibility. Figure 2 in Section 2 represents comparable information in a condensed form for increased legibility.
