# Supplementary figures and images for: Microfabrication Process Development for a Polymer-Based Lab-on-Chip Concept Applied in Attenuated Total Reflection Fourier Transform Infrared Spectroelectrochemistry
Source: Sensors (Basel). 2023 Jul 8;23(14):6251. doi: 10.3390/s23146251 (PMC10383751; doi:10.3390/s23146251)

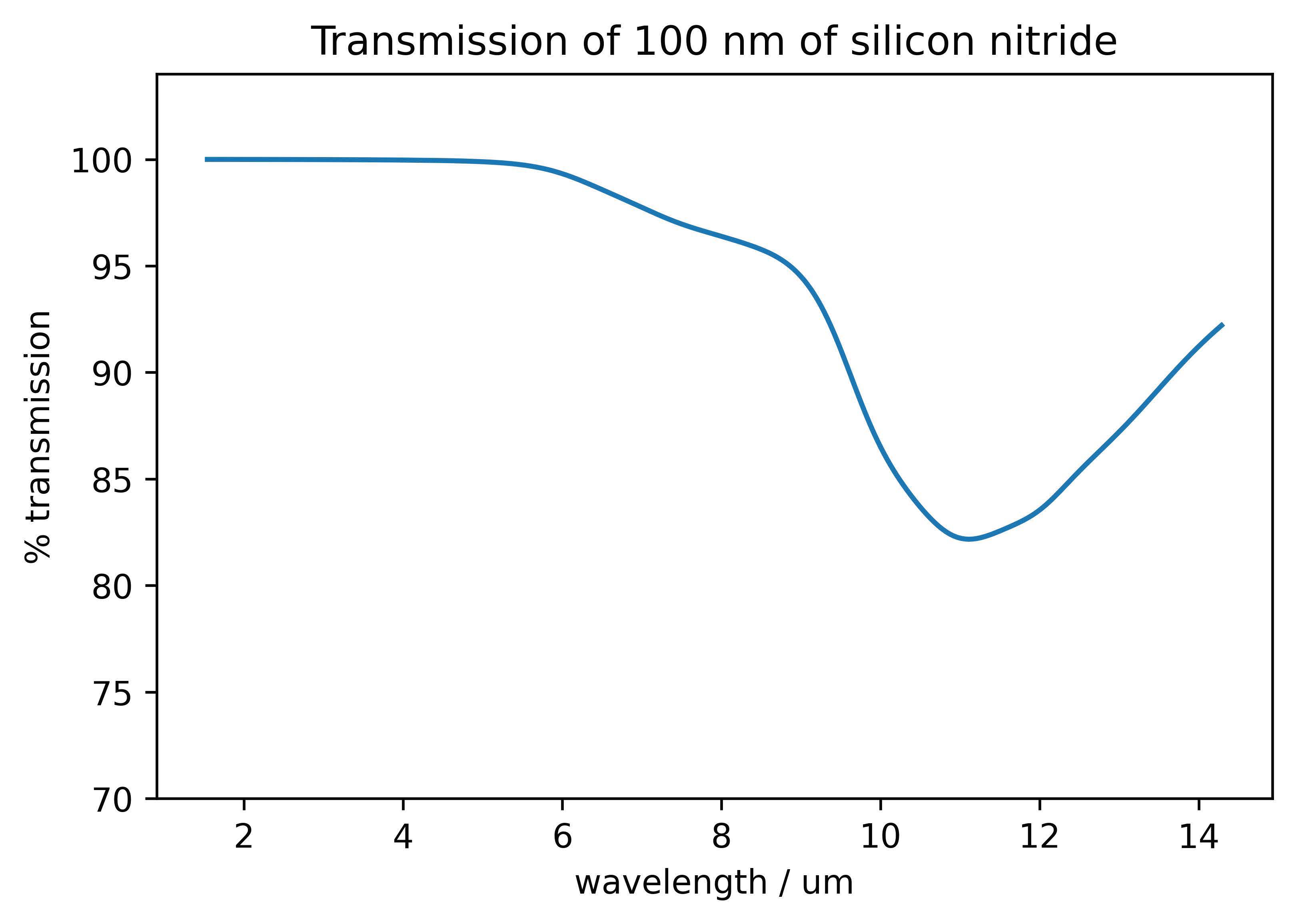

Supplement: Supplementary file 1 [file sensors-23-06251-s001.zip › sensors-2467751-supplementary.jpg]
